# Supplementary figures and images for: Biocompatibility of Small-Diameter Vascular Grafts in Different Modes of RGD Modification
Source: Polymers (Basel). 2019 Jan 18;11(1):174. doi: 10.3390/polym11010174 (PMC6401695; doi:10.3390/polym11010174)

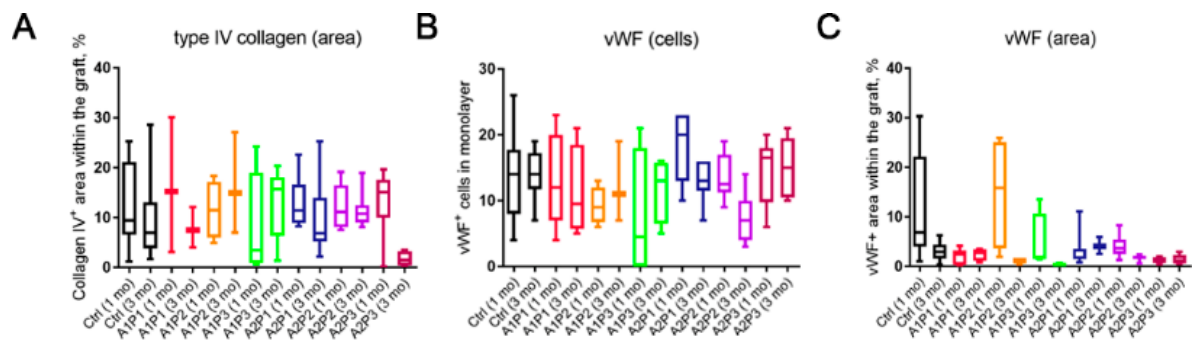

Supplement: Supplementary file 1 [file polymers-11-00174-s001.pdf]
